# Immunotherapy for ocular melanoma: a bibliometric and visualization analysis from 1991 to 2022

**DOI:** 10.3389/fonc.2023.1161759

**Published:** 2023-05-31

**Authors:** Yao Tan, Yijie Lu, Sheng Chen, Chang Zou, Bo Qin

**Affiliations:** ^1^ Shenzhen Aier Eye Hospital, Aier Eye Hospital, Jinan University, Shenzhen, China; ^2^ Shenzhen Eye Hospital, Shenzhen Key Laboratory of Ophthalmology, Affiliated Hospital of Jinan University, Shenzhen, China; ^3^ Shenzhen Aier Ophthalmic Technology Institute, Shenzhen, China; ^4^ School of Life and Health Sciences, The Chinese University of Kong Hong, Shenzhen, Guangdong, China

**Keywords:** ocular melanoma, uveal melanoma, immunotherapy, bibliometric, CiteSpace, VOSviewer

## Abstract

**Background:**

In recent years, new therapeutic options to overcome the mechanisms of tumor immune suppression be effective in the treatment of cutaneous melanoma. These approaches have also been applied in ocular melanoma. The aim of this study is to present the current status and research hotspots of immunotherapy for ocular melanoma from a bibliometric perspective and to explore the field of immunotherapy for malignant ocular melanoma research.

**Methods:**

In this study, the Web of Science Core Collection database (WoSCC) and Pubmed were selected to search the literature related to immunotherapy of ocular melanoma. Using VOSviewer, CiteSpace, the R package “bibliometrix,” and the bibliometric online platform through the construction and visualization of bibliometric networks, the country/region, institution, journal, author, and keywords were analyzed to predict the most recent trends in research pertaining to ocular melanoma and immunotherapy.

**Results:**

A total of 401 papers and 144 reviews related to immunotherapy of ocular melanoma were included. The United States is the main driver of research in the field, ranking first in terms of the number of publications, total citations, and H-index. The UNIVERSITY OF TEXAS SYSTEM is the most active institution, contributing the most papers. Jager, Martine is the most prolific author, and Carvajal, Richard is the most frequently cited author. CANCERS is the most published journal in the field and J CLIN ONCOL is the most cited journal. In addition to ocular melanoma and immunotherapy, the most popular keywords were “uveal melanoma” and “targeted therapy”. According to keyword co-occurrence and burst analysis, uveal melanoma, immunotherapy, melanoma, metastases, bap1, tebentafusp, bioinformatics, conjunctival melanoma, immune checkpoint inhibitors, ipilimumab, pembrolizumab, and other research topics appear to be at the forefront of this field’s research and have the potential to remain a hot research topic in the future.

**Conclusion:**

This is the first bibliometric study in the last 30 years to comprehensively map the knowledge structure and trends in the field of research related to ocular melanoma and immunotherapy. The results comprehensively summarize and identify research frontiers for scholars studying immunotherapy associated with ocular melanoma.

## 1 Introduction

Melanoma is a relatively rare tumor originating from melanocytes in the skin, mucous membranes (nasal mucosa, oropharynx, lungs, gastrointestinal tract, vagina, anus/rectum, urinary tract), and eyes (uvea, conjunctiva, eyelids, orbit), while melanomas of the ocular choroid, ciliary body, and iris are collectively referred to as uveal melanoma (1–3). Ocular melanoma is the second most common type after cutaneous melanoma and is the most common primary intraocular malignancy in adults (4). The vast majority of ocular melanomas originate in the uvea, accounting for 82.5% of all melanomas, while the incidence of conjunctival melanoma is lower (5). In the United States, the annual incidence of ocular melanoma is approximately 6 per million, with a male predominance and a male-to-female ratio of 1.29. The incidence of uveal melanoma and conjunctival melanoma is 4.9 per million and 0.4 per million, respectively (2, 6–8). Important cytogenetic and genetic risk factors for melanoma development include chromosome 3 haplogroups, guanine mutations in the nucleotide-binding protein GNAQ/GNA11, and braca1-associated protein 1 (BAP 1) deletion (9–11). Both ocular removal and treatment that maintains the eye are options for localized ocular melanoma. The treatment strategy for ocular melanoma should be individualized. For primary uveal melanoma, plaque brachytherapy is currently the most commonly used eye-preserving treatment for small to medium-sized uveal melanomas. Proton beam radiation therapy is indicated for tumors of significant size, challenging shape, and location, while removal of the eye is limited to advanced tumors (12, 13). Nearly 50% of patients with uveal melanoma get metastases within 15 years of initial diagnosis, despite high local disease control rates with surgery or radiation therapy (14). Conjunctival melanoma is rare, but its incidence is on the rise. It occurs mainly in white adults. Conjunctival melanoma treatment currently involves significant local excision, adjuvant therapy with brachytherapy, cryotherapy, and local application of chemotherapeutic drugs (15, 16). For the treatment of conjunctival melanoma, extensive local surgical resection followed by individualized proton beam radiotherapy (PBRT) is increasingly becoming a feasible strategy, even in locally advanced conjunctival melanoma (17). Either uveal melanoma or conjunctival melanoma, patients with metastases have a poor prognosis because there are no effective systemic treatments available (18–20). The greatest success in the treatment of cutaneous melanoma has been achieved in recent years (21), with immunotherapy with anti-ctla -4 and anti-pd -1/PD-L1 drugs significantly changing the treatment paradigm for metastatic cutaneous melanoma (22, 23). However, as a subtype of malignant melanoma, the significant progression-free survival (PFS) and overall survival (OS) of immunotherapy and molecularly targeted agents for advanced non-uveal melanoma have not been matched in ocular melanoma (24, 25). As our understanding of the genetic and molecular pathways of ocular melanoma pathogenesis has advanced considerably, clinical trials of immunotherapy for ocular melanoma have been completed or are underway, and data from these well-designed studies will help guide the future direction of this rare disease. Thus, immunotherapy of ocular melanoma has important research value and broad clinical applications.

Bibliometric analysis is a powerful tool for understanding the research landscape of a field of study. It uses quantitative methods to measure and analyze the impact of scholarly work. This can include the number of citations, the number of authors and institutions, the number of journals, and other data. Bibliometric analysis can provide insight into trends, patterns, and relationships in research, helping to identify key areas of focus and potential opportunities for further research (26, 27). There are no bibliometric studies on immunotherapy of ocular melanoma. The purpose of this paper is to systematically summarize and visually analyze the literature in the field of immunotherapy of ocular melanoma based on Web of Science and Pubmed, using CiteSpace and VOSviewer software, to depict the research hotspots and trends of immunotherapy of ocular melanoma in the past 30 years.

## 2 Materials and methods

### 2.1 Data source and search strategy

**Figure 1:**
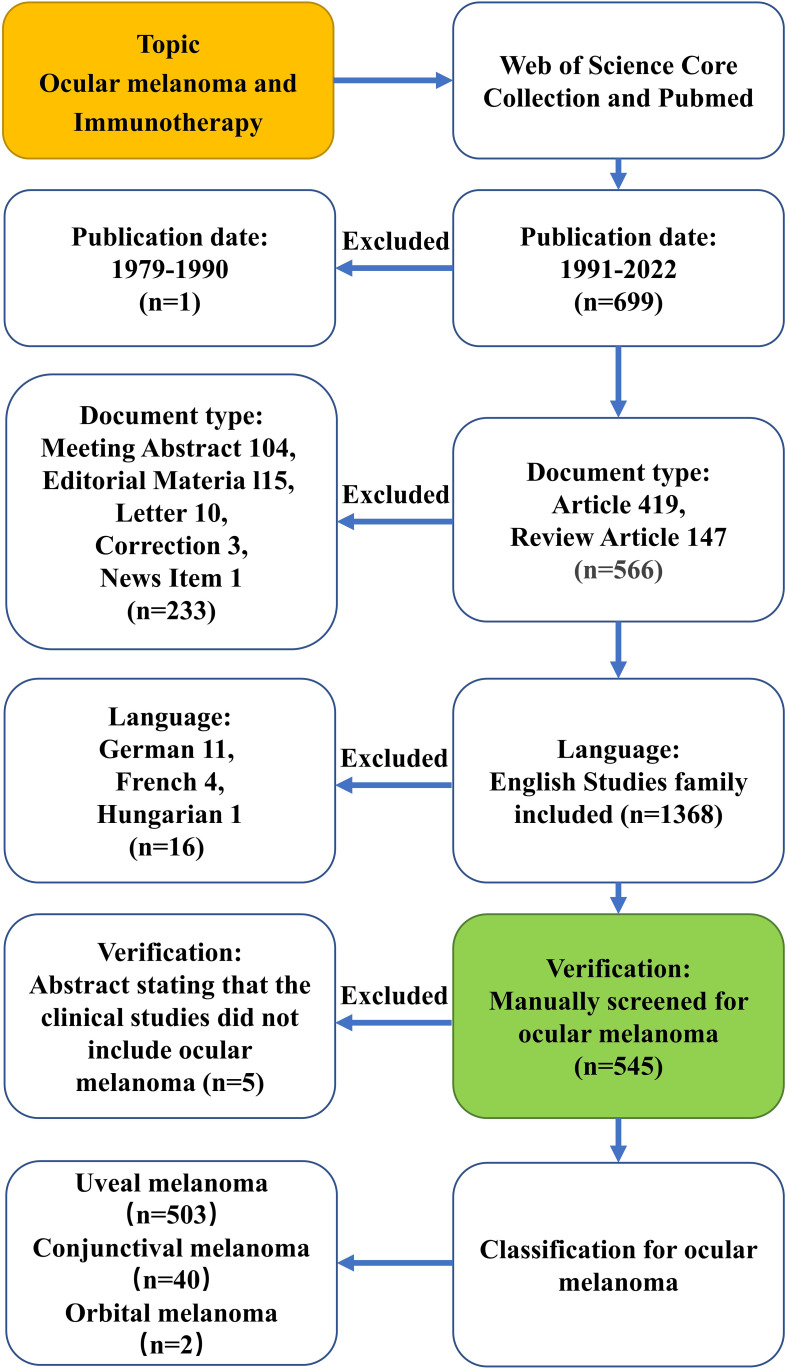
Process flow diagram for the selection and search of the literature.

Considering the quality of eligible literature and the appropriate reference format requirements, the Web of Science Core Collection (WoSCC, Clarivate Analytics) is the most suitable database for bibliometric analysis based on previous studies (28, 29). PubMed is the MEDLINE database, which is one of the most authoritative databases of abstract-based medical literature in the world today and the most widely used free MEDLINE search tool on the Internet. These two databases are classic citation databases that include literature abstracts and other relevant data, such as citations and research collaboration information, which are useful for bibliometric analysis. Furthermore, they can directly inform the construction and visualization of bibliometric networks in VOSviewer and CiteSpace. Therefore, these two databases were selected for this investigation. Ocular melanoma is the second most common type of melanoma after skin, originating from melanocytes in the conjunctiva and uvea of the eye. Although rare, it can also arise from melanocytes located in the orbit. The vast majority of ocular melanomas originate in the uvea, while the incidence of conjunctival melanoma is much lower. In contrast, uveal melanoma originates from the melanocytes of the uvea, including the iris, ciliary body, and choroid, and is the most common primary intraocular malignancy in adults. There were only a few articles about the immunotherapy of ocular melanoma before 1991, so every literature search was conducted on the same day (19 October 2022) to prevent the bias caused by database updates. Details of the search strategy are provided in **Supplementary Material** **Supplementary Data**. Terms related to immunotherapy and ocular melanoma entered into the WoS engine were extracted from the Medical Subject Headings (MeSH) in PubMed, and the wildcard “*” was used in place of any number of characters for the most comprehensive search of relevant literature. The use of truncation searches and the truncation symbol “*” increased recollection and prevented missed examination. The criteria for selecting literature were as follows: (1) The time period covered the period from January 1, 1991, through October 19, 2022; (2) Only reviews and articles were available as documents; (3) Language could only be set in English. Keep track of every piece of content, including the title, authors, abstract, keywords and cited references. 699 articles in total about the immunotherapy of ocular melanoma were searched from 1991 to 2022 (October 19, 2022). 149 publications were excluded including meeting abstract, editorial material, letter, correction, news item and non-English works of literature. Furthermore, the remaining 550 papers were manually re-screened to exclude 5 papers that were not related to ocular melanoma. The final dataset, which consisted of 545 legitimate literatures (401 articles and 144 reviews), was eventually obtained and exported in plain text format using the “Full Record and Cited References” function for further analysis. The text files were renamed to “download.txt,” which CiteSpace software was able to read them. **Figure 1** depicts the comprehensive literature screening procedure. The above data was imported into Microsoft Excel 365 for further analysis. To guarantee the accuracy of the findings, data extraction, literature selection and analysis were all carried out separately by two researchers. Any disagreements between the two reviewers were resolved by conversation with an experienced expert.

### 2.2 Bibliometric analysis and visualization

In this analysis, the primary factors considered were annual publications, citation count, country/region, journal, institutions, authors, co-cited references, and keywords. The H-index is used to measure the production and influence of a nation, institution, or journal. It is determined by taking into account that a scientist/country publishes h papers, each of which receives at least h citations (30). The H-index is a valuable metric for researchers because it allows them to see the impact of their work. Five bibliometric tools were used in this work to further analyze the information acquired from the aforementioned sources, including the software CiteSpace (6.3.R3), VOSviewer (1. 6. 18), Bibliometrics (3.1.4), Microsoft Office Excel 365, “bibliometrix” R package(version 3.1.4), and an online platform (https://bibliometric.com/).

CiteSpace, a java-based bibliometric tool developed by Prof. Chen Chaomei of Drexel University, is an influential visualization software for obtaining quantitative information and discovering relevant trends and dynamics in specific scientific fields (31). Its citation and keyword burst detection identify a dramatic increase in scientific activity over a limited period and captures the growing interest in a specific research area (32). The citespace software settings are as follows: (1) Time slicing: 1991-2022; time zone selection (year per slice): 1 year; node type: reference, keyword. (2) The threshold (g-index): 10, that is, the g index is the largest number that equals the average number of citations of the most highly cited g publications. In order to prevent the co-citation network from being too complex, a Pathfinder algorithm was used in this paper, which could simplify the network by removing the edges that violate the triangle inequality and accurately extract the key structure of the network. The default system was selected for visualization. CiteSpace standardized algorithms included the Cosine similarity algorithm, Jaccard similarity algorithm, and Dice similarity coefficient. The next step was to analyze the relationship between the data. The software used the method of cluster analysis to reveal the correlation. CiteSpace clustering algorithm mainly used nominal terms to detect research hotspots, which could help researchers find mutation words in the map, explore research hotspots, and grasp the research direction. There were three Clustering algorithms: Clustering algorithm, LLR algorithm, and MI algorithm. At the same time, OALM was used to analyze the number of common national articles by year, the number of common keywords by year, partnerships (including authors, institutions, and countries) and article citation relationships.

VOSviewer is a software tool widely used to visualize and build bibliometric network maps, developed by Professors Eck and Waltman at Leiden University using the Java language. It can evaluate and visualize research characteristics from different perspectives and has powerful features for constructing and visualizing bibliometric networks such as co-authorship, co-citation, and co-occurrence network maps for journals, countries, authors, or keywords (33). In addition, VOSviewer can provide three types of network maps, including network visualization maps, coverage visualization maps, and density visualization maps (34). The VOSviewer software package enables construction and analysis of bibliometric maps. In this study, the parameters of the VOSviewer were as follows: The counting method was selected for “full counting.” The minimum number of citations for the co-cited authors and co-cited references was twenty and forty, respectively. The unit of analysis of the co-occurrence keyword was “all keyword,” and the threshold for the minimum number of occurrences was set to thirty. Each software allows for the construction and visualization of bibliometric networks to facilitate understanding of the GM/CI research. Specifically, the distribution of each component analyzed in the bibliometric analysis was assessed by a software package applying machine learning. For this, we used the following variables: annual scientific production, average citations per year, most relevant journals, journals dynamics, most impact journals by H-index or total citations (TC), top journals’ production over time, most relevant authors, top authors’ production over time, author local impact, most relevant affiliations, relevant funding agencies, country scientific production, collaboration network of countries, corresponding author’s country, top countries’ production over time, historical direct citation network, most global cited papers, most relevant keywords and cluster analysis of keywords. The journals’ impact factor (IF) and partition refer to the “2020 Journal Citation Reports”. In addition, Microsoft Office Excel 365 was used for index model building, while the “bibliometrix” R package was used for local citation statistics. In this descriptive study, variables were presented as numbers and percentages. No comparisons were made; therefore, no P values was set.

## 3 Materials and methods ethics statement

All of the data utilized in this research were sourced from open-access databases, and neither human participants nor animals were employed in this investigation. As a result, ethical approval wasn’t necessary.

## 4 Results

### 4.1 General trends in paper publication

**Figure 2:**
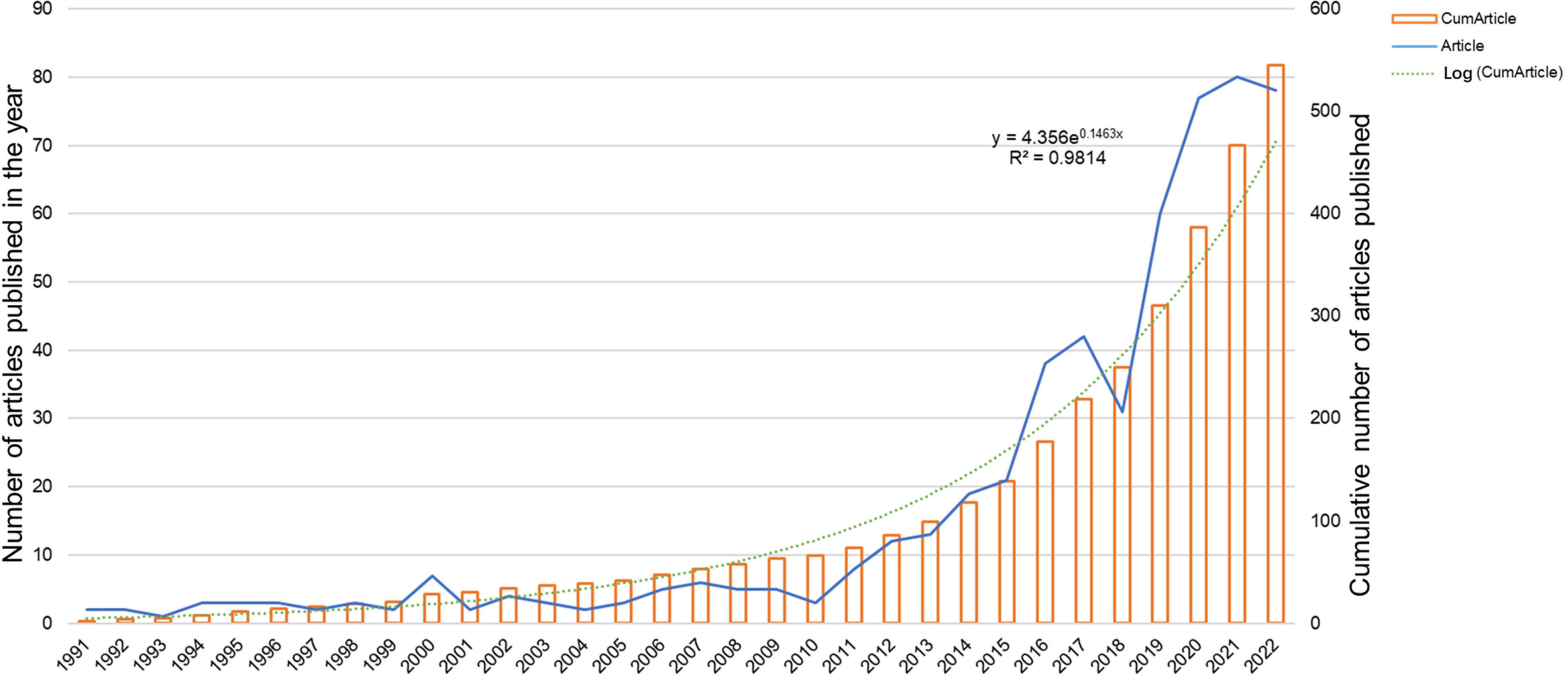
Global trend of annual publications related to immunotherapy for ocular melanoma from 1991 to 2022.

Quantitative analysis of published papers can help identify which papers are most influential and useful in the scientific community. This information can be used to improve the quality of scientific papers and to guide the research of scientists. The distribution of publications in the literature for each year from 1991-2022 is shown in **Figure 2**. The annual number of publications on immunotherapy for ocular melanoma gradually increased during the 31 years, except for the decreasing number of publications at individual time points. From 1991 to 2010, there were fewer than 8 publications per year on average, and starting in 2011, the number of publications gradually entered a rapid growth phase. A logistic regression model was used to plot the time curve of the number of publications and predict the future global trend of the number of publications. The figure shows the fitted curve of the annual publication trend with a correction factor R^2^ of 0.9814 (y=4.365e^0.1463x^). In conclusion, these results indicate that the research related to ocular melanoma and immunotherapy has attracted more researchers’ attention and entered a phase of rapid development. These findings suggest that research on immunotherapy and ocular melanoma has gained more traction among scientists and has entered a phase of continuous growth.

### 4.2 Analysis of published articles by countries/regions

**Figure 3:**
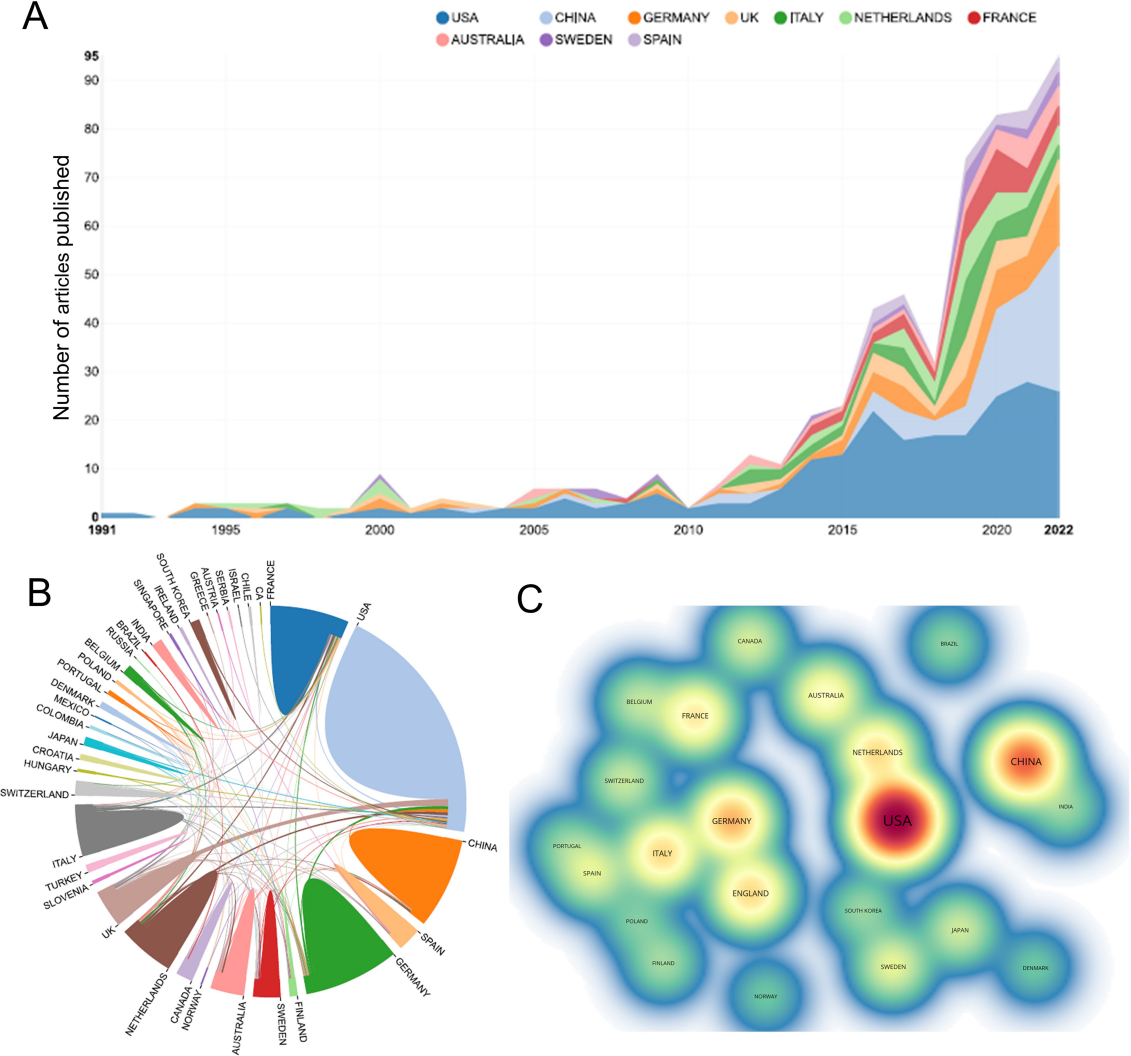
**(A)** The annual number of publications in the top 10 countries/regions from 1991 to 2022. **(B)** The network map of collaboration between countries/regions based on the website https://bibliometric.com/. **(C)** The density map of countries/regions based on VOSviewer. Minimum number of documents of a country ≥ 5.

A total of 545 papers from 218 academic journals with 3266 authors from 982 institutions in 42 countries/regions were included in this study. The top 10 countries/regions in terms of the number of articles published in this research area were mainly the United States (228, 41.83%), China (94, 17.25%), Germany (59, 10.83%), and England (43, 7.89%). The total citations were 7499, 1010, 2050, and 1687, while the corresponding H-index were 51, 15, 22, and 22. Regarding citation frequency, the Spanish literature had the highest average citation frequency (43.31). England had the second-highest average frequency of citations (39.23), followed by France (37.97), Australia (37.79), and Germany (34.75) (**Table 1**). The annual trend in the number of papers is shown in **Figure 3A**, with the United States being the leading country in the number of annual documents from 1991 to 2022. A collaboration analysis was conducted to examine the international collaborations observed from 1998-2022. **Figure 3B** shows that the U.S. has the most international collaborations in this area, followed by France. And England has the most substantial ties to the United States. The United States is the largest node on the national network map (**Figure 3C**). In addition, certain countries, such as China, Germany, and England, show high centrality, which implies that these countries may play a crucial role in this research area.

**Table 1:** The top 10 countries/regions contributing to immunotherapy for ocular melanoma.

Rank	Countries	Article count	Percentage	H-index	Total citations	Average citation per article
1	USA	228	41.83%	51	7,499	32.89
2	CHINA	94	17.25%	15	1,010	10.74
3	GERMANY	59	10.83%	22	2,050	34.75
4	ENGLAND	43	7.89%	22	1,687	39.23
5	NETHERLANDS	43	7.89%	21	1,399	32.53
6	ITALY	43	7.89%	21	1,353	31.47
7	FRANCE	36	6.61%	18	1,367	37.97
8	AUSTRALIA	29	5.32%	16	1,096	37.79
9	SWEDEN	18	3.30%	11	541	30.06
10	SPAIN	17	3.12%	11	738	43.41

### 4.3 Analysis of institutions

**Figure 4:**
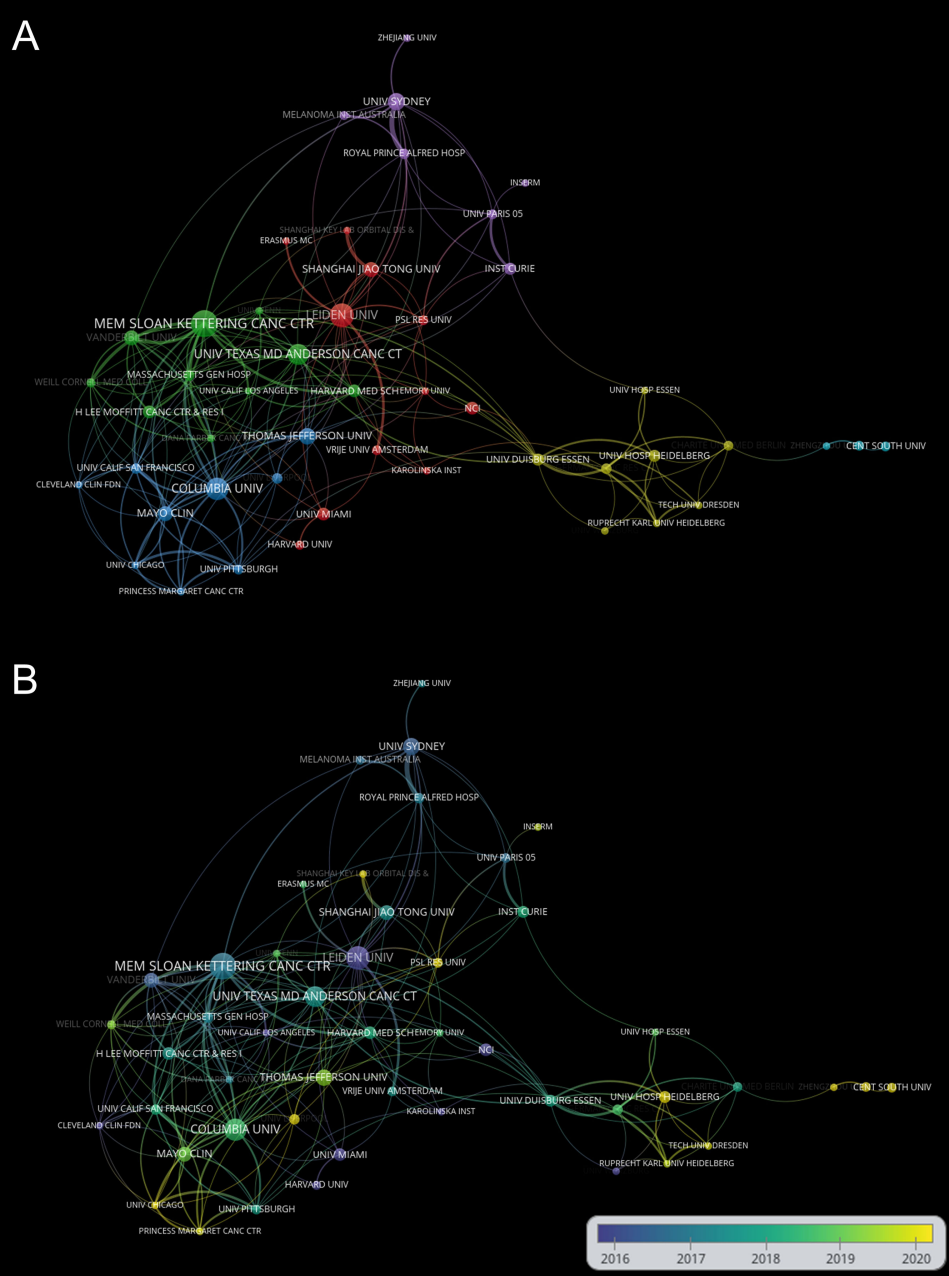
**(A)** Network visualization of institutions based on VOSviewer. **(B)** Visualization of institutions overlays based on VOSviewer. Nodes marked in purple or blue represent institutions that appeared earlier and started to be studied, while those marked in yellow represent institutions that are emerging. Minimum number of documents of an organization ≥ 5.

The top 10 institutions in the ranking of publications are shown in **Table 2**. The top 10 institutions published 290 articles (53.21%), among which the UNIVERSITY OF TEXAS SYSTEM published the most articles with 36 (6.6%), followed by MEMORIAL SLOAN KETTERING CANCER CENTER with 32 (5.9%) and LEIDEN UNIVERSITY with 31 (5.7%). The analysis of the collaborative network of institutions is shown in **Figure 4A**. Literature from 982 institutions was included, with no less than 5 articles per institution. The data were analysed using VOSviewer with 48 nodes, 6 clusters, and 201 links on the network map, with MEMORIAL SLOAN KETTERING CANCER CENTER as the node center. **Figure 4B** shows the time overlay into the analysis of the cooperative network of the institution. In this map, the node colors reflect the average year of emergence corresponding to each institution. The top 10 institutions joined earlier, as shown by the color gradient in the lower right corner.

**Table 2:** The top 10 institutions with the most publications on immunotherapy for ocular melanoma.

Rank	Institutions	Countries	Article count	H-index	Total citations	Average citation per article
1	UNIVERSITY OF TEXAS SYSTEM	USA	36	20	1,549	43.03
2	MEMORIAL SLOAN KETTERING CANCER CENTER	USA	32	20	1,900	59.38
3	LEIDEN UNIVERSITY	NETHERLANDS	31	17	927	29.9
4	UDICE FRENCH RESEARCH UNIVERSITIES	FRANCE	31	16	1,232	39.74
5	LEIDEN UNIVERSITY MEDICAL CENTER	NETHERLANDS	30	16	893	29.77
6	HARVARD UNIVERSITY	USA	28	18	1,569	56.04
7	UTMD ANDERSON CANCER CENTER	USA	27	15	1,143	42.33
8	UNICANCER	FRANCE	26	14	887	34.12
9	COLUMBIA UNIVERSITY	USA	25	14	1,108	44.32
10	LEIDEN UNIVERSITY EXCL LUMC	NETHERLANDS	24	15	828	34.5

### 4.4 Analysis of authors and co-cited authors

The number of research papers published by the authors reflects their contribution to the research in the field. The top 10 authors published 114 papers, accounting for approximately 20.91% of the total number of papers published in the field. The top 10 most prolific authors in the field are listed in **Table 3**. The most prolific authors were Jager, Martine [20 (3.67%)], followed by Carvajal, Richard [17 (3.12%)], Patel, Sapna P. [10 (1.83%)], Heppt, Markus [10 (1.83%)] and Berking, Carola [10 (1.83%)]. **Table 4** also lists the 10 most frequently co-cited authors, including Carvajal, Richard (386), Jager, Martine (211), Sullivan, R. J (207), Piulats, Josep M (205) Postow, Michael A. (199). Carvajal, Richard (COLUMBIA UNIVERSITY, USA), Jager, Martine (LEIDEN UNIVERSITY MEDICAL CENTER, NETHERLANDS), Heppt, Markus, and Berking, Carol (FRIEDRICH ALEXANDER UNIVERSITY, GERMANY) are among the top 10 authors in both lists.

**Table 3:** The top 10 most productive authors contributed to immunotherapy for ocular melanoma.

Rank	Author	Article count	H-index	Countries	Total citations	Average citation per article
1	Jager, Martine	20	13	NETHERLANDS	722	36.10
2	Carvajal, Richard	17	12	USA	1,083	63.71
3	Patel, Sapna P.	10	8	USA	185	18.50
4	Heppt, Markus	10	6	GERMANY	322	32.20
5	Berking, Carola	10	6	GERMANY	322	32.20
6	Piperno-Neumann, Sophie	10	6	FRANCE	382	38.20
7	Hassel, Jessica C.	10	5	GERMANY	221	22.10
8	Jia, Renbing	9	6	CHINA	258	28.67
9	Orloff, Marlana	9	6	USA	310	34.44
10	Hwu, Patrick	9	8	USA	214	23.78

**Table 4:** The top 10 co-cited authors with the most publications on immunotherapy for ocular melanoma.

Rank	Co-cited author	Local Citations	Countries	Institutions
1	Carvajal, Richard	386	USA	COLUMBIA UNIVERSITY
2	Jager, Martine	211	NETHERLANDS	LEIDEN UNIVERSITY MEDICAL CENTER
3	Sullivan, R. J.	207	USA	HARVARD MEDICAL SCHOOL
4	Piulats, Josep M	205	SPAIN	CATALAN INSTITUTE OF ONCOLOGY
5	Postow, Michael A.	199	USA	MEMORIAL SLOAN KETTERING CANCER CENTER
6	Shoushtari, A. N.	192	USA	MEMORIAL SLOAN KETTERING CANCER CENTER
7	Ott, Patrick Alexander	175	USA	HARVARD MEDICAL SCHOOL
8	Utikal, Jochen Sven	169	GERMANY	RUPRECHT KARL UNIVERSITY OF HEIDELBERG
9	Berking, Carola	140	GERMANY	FRIEDRICH ALEXANDER UNIVERSITY
10	Heppt, Markus	140	GERMANY	FRIEDRICH ALEXANDER UNIVERSITY

### 4.5 Analysis of journals and co-cited journals

**Figure 5:**
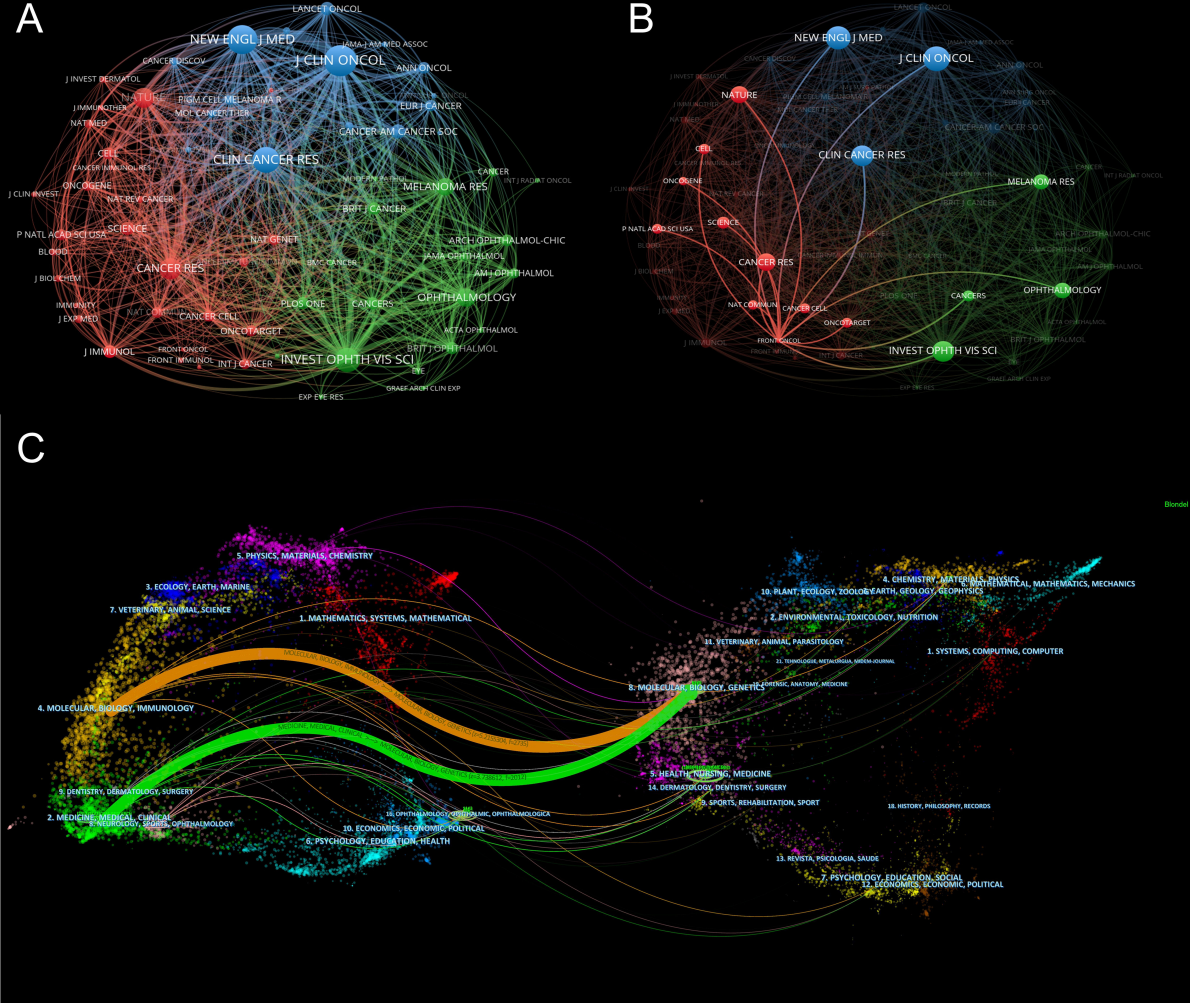
**(A)** Network map of co-citation analysis of journals based on VOSviewer. Minimum number of citations of a source ≥ 200. **(B)** Network map of co-citation analysis of FRONTIERS IN ONCOLOGY based on VOSviewer. **(C)** The dual-map overlay of academic journals in the field of immunotherapy of ocular melanoma based on CiteSpace software.

In this research field, the collected material was published in 218 journals altogether. The top 10 journals published 172 papers related to immunotherapy of ocular melanoma, accounting for 31.56% of the total number of publications. The top 10 journals with the highest output in this study are listed in **Table 5**. The journal with the highest number of publications was CANCERS (IF=6.575,2021) [51 (9.36%)], followed by MELANOMA RESEARCH (IF=3.199,2021) [29 (5.32%)], FRONTIERS IN ONCOLOGY (IF=5.738,2021) [15 (2.75%)] and JOURNAL FOR IMMUNOTHERAPY OF CANCER (IF=12.469,2021) [15 (2.75%)]. Among the top 10 journals, the JOURNAL FOR IMMUNOTHERAPY OF CANCER has the highest IF (12.469). Co-citation analysis was performed by VOSviewer identified journals with a citation frequency ≥ 200 (**Figure 5A**). **Figure 5B** shows the co-citation analysis of FRONTIERS IN ONCOLOGY. The co-citation analysis measures the degree of association between articles. The co-citation analysis illustrates the relationship between items based on the number of times they are cited together. The influence of a journal depends on its co-citation frequency. The top 5 co-cited journals in order were: J CLIN ONCOL (1514), NEW ENGL J MED (1369), CLIN CANCER RES (1170), INVEST OPHTH VIS SCI (1143), and CANCER RES (925). In addition, a dual map overlay of journals related to immunotherapy for ocular melanoma was constructed (**Figure 5C**). The dual map overlay of journals depicted the subject distribution of academic journals, and the left-to-right sample waves depicted the citation associations, represented by colored paths. There are two main citation paths on the current map. The two main citation paths are marked in orange and green in **Figure 5C**. The two main paths show that literature published in molecular/biology/immunology and medicine/medical/clinical is mainly cited by researchers published in molecular/biology/genetics journals.

**Table 5:** The top 10 journals and co-cited journals related to immunotherapy for ocular melanoma.

Rank	Journals	Counts	Total citations	Average citation per article	IF	Co-cited Journals	Total co-citations	IF
1	CANCERS	51	612	12.00	6.575	J CLIN ONCOL	1514	50.717
2	MELANOMA RESEARCH	29	675	23.28	3.199	NEW ENGL J MED	1369	176.079
3	FRONTIERS IN ONCOLOGY	15	121	8.07	5.738	CLIN CANCER RES	1170	13.801
4	JOURNAL FOR IMMUNOTHERAPY OF CANCER	15	517	34.47	12.469	INVEST OPHTH VIS SCI	1143	4.925
5	INVESTIGATIVE OPHTHALMOLOGY VISUAL SCIENCE	14	429	30.64	4.925	CANCER RES	925	13.312
6	EUROPEAN JOURNAL OF CANCER	12	411	34.25	10.002	NATURE	873	69.504
7	CANCER IMMUNOLOGY IMMUNOTHERAPY	11	341	31.00	6.63	OPHTHALMOLOGY	761	14.277
8	FRONTIERS IN IMMUNOLOGY	10	107	10.70	8.786	MELANOMA RES	722	3.199
9	ONCOTARGET	8	192	24.00	0	CANCER-AM CANCER SOC	525	6.921
10	BMC CANCER	7	139	19.86	4.638	SCIENCE	492	63.714

### 4.6 Analysis of references with citation burst

**Figure 6:**
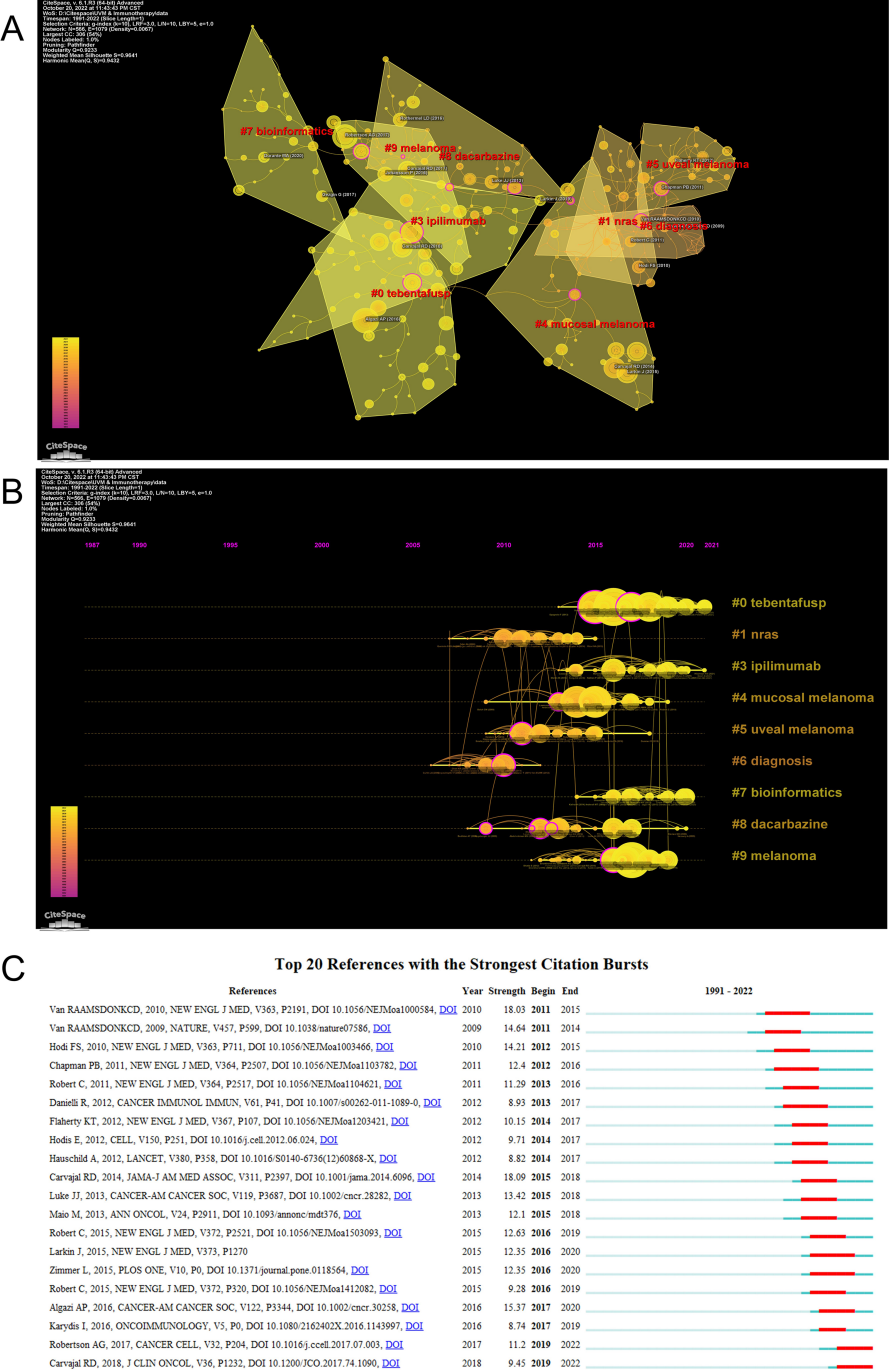
**(A)** The clustered network map of co-cited references using by CiteSpace. **(B)** The timeline view of clusters of co-cited references using by CiteSpace. **(C)** The top 20 references with the strongest citation bursts. The red bar represents the begin and end year of the burst duration.

CiteSpace can divide the co-citation network into clusters, displaying closely related references in one cluster and loosely connected references in another cluster. The words of the citation titles in the clusters are used to denote each cluster. The nine largest clusters extracted from references in the cited literature include #0 tebentafusp, #1 nras, #3 ipilimumab, #4 mucosal melanoma, #5 uveal melanoma, #6 diagnosis, #7 bioinformatics, #8 dacarbazine, and #9 melanoma (**Figure 6A**), with cluster 2 not shown and unfiltered because there were fewer than 10 articles. Cluster plots can be converted to a timeline format using the cluster numbers as the y-axis, which reflects the temporal characteristics of the research hotspots in the field (**Figure 6B**). The timeline plot depicts the research progress of the field and its eight subfields over time (**Table S1**). The largest cluster is #0 tebentafusp, followed by #9 melanoma and #4 mucosal melanoma. #6 diagnosis and #1 nras occur earlier, suggesting that early development of ocular melanoma is focused on a definitive diagnosis. #0 tebentafusp and #3 ipilimumab are current research hotspots, suggesting that these two agents are currently favorable for immunotherapy of ocular melanoma. In addition, citation burst is a valuable indicator of references that reflect the interest of researchers in a particular field over some time. **Table 6** lists the top 10 most cited original publications on immunotherapy for ocular melanoma. These selected articles cover the years 2009 to 2020. The most frequently cited paper was published in 2015 and was written by Dirk Schadendorf with 306 citations (38.25). Tadepally Lakshmikanth’s paper was the second-most cited, receiving 249 citations (17.79). Heinz Läubli’s third co-cited paper was published and received 212 citations (26.50). In our study, CiteSpace identified the top 20 most frequent bursts, as shown in **Figure 6C**, where the blue line indicates the period and the red line indicates the duration of the reference burst occurring. The publication entitled “Effect of selumetinib vs chemotherapy on progression-free survival in uveal melanoma: a randomized clinical trial” published in 2014 ranked first (intensity = 18.09).

**Table 6:** The top 10 documents with the most citations of immunotherapy for ocular melanoma.

Rank	Paper	DOI	Total	TC per Year	Normalized TC
			Citations		
1	SCHADENDORF D, 2015, NAT REV DIS PRIMERS	10.1038/nrdp.2015.3	306	38.25	5.44
2	LAKSHMIKANTH T, 2009, J CLIN INVEST	10.1172/JCI36022	249	17.79	2.64
3	LAUBLI H, 2015, J IMMUNOTHER CANCER	10.1186/s40425-015-0057-1	212	26.50	3.77
4	ALGAZI AP, 2016, CANCER-AM CANCER SOC	10.1002/cncr.30258	209	29.86	5.57
5	JAGER MJ, 2020, NAT REV DIS PRIMERS	10.1038/s41572-020-0158-0	172	57.33	8.73
6	YANG J, 2018, THER ADV MED ONCOL	10.1177/1758834018757175	158	31.60	4.19
7	CABEL L, 2017, ANN ONCOL	10.1093/annonc/mdx212	156	26.00	4.27
8	ZIMMER L, 2015, PLOS ONE	10.1371/journal.pone.0118564	155	19.38	2.76
9	JOVANOVIC P, 2013, INT J CLIN EXP PATHO	NA	147	14.70	2.57
10	LUKE JJ, 2013, CANCER-AM CANCER SOC	10.1002/cncr.28282	143	14.30	2.50

To better understand the various subtypes of ocular melanoma, we performed separate cluster analyses for conjunctival melanoma as well as orbital melanoma. Orbital melanoma had only two publications and could not be analyzed. Conjunctival melanoma has 40 publications. As shown in **Figure S1**, the four largest clusters extracted from the cited literature include #1 cutaneous melanoma, #2 immunology, #3 braf mutation, and #4 pd-1. The largest cluster that is also a current research hotspot is #2 immunology, while #3 braf mutation occurs earliest, suggesting that the early development of conjunctival melanoma is mainly focused on its biological studies. The #4 pd-1 shows that this antibody drug contributes to the immunotherapy of conjunctival melanoma.

### 4.7 Analysis of keywords and hotspots

**Figure 7:**
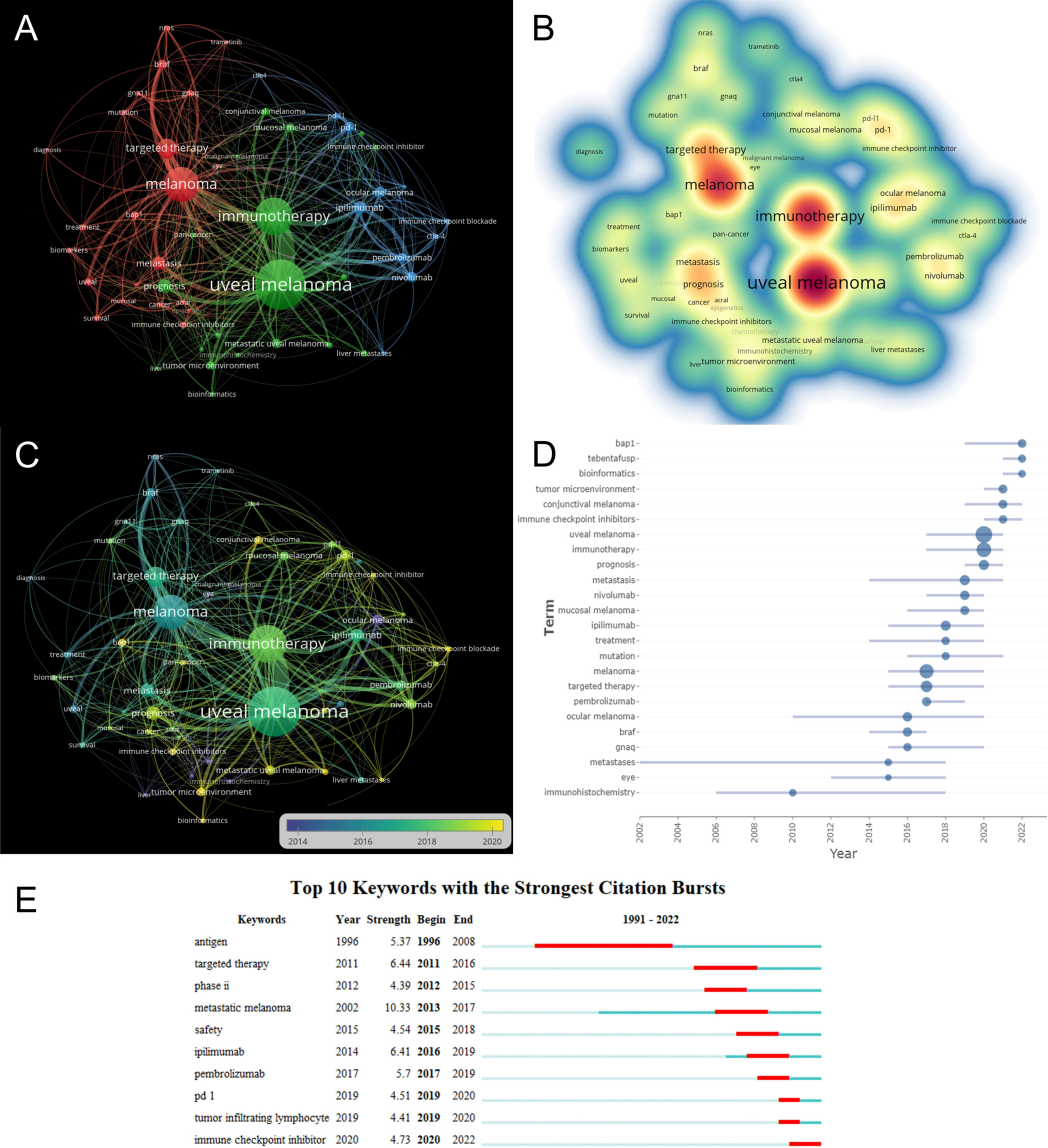
**(A)** VOSviewer visualization map of co-occurrence keywords. **(B)** Density visualization of keywords based on VOSviewer. **(C)** Overlay visualization of keywords based on VOSviewer. Nodes marked in purple or blue represent earlier appearing keywords, while nodes marked in yellow represent current hot keywords. Minimum number of occurrences of keywords ≥ 5. **(D)** The frequency of top 24 keywords over time. **(E)** The top 10 keywords with the strongest citation bursts.

Keywords can offer researchers information on research topics and research methodologies of publications, and keyword co-occurrence analysis is frequently used to identify research hotspots and directions in this field of study. The co-occurrence analysis illustrates the relationship between the items, based on the number of works in which they appear together, which is one of the important means to track scientific development. A network visualization map is generated for keywords with more than 5 co-occurrences. As shown in **Figure 7A**, there are 53 nodes on the visualization map, among which “uveal melanoma” is in the center of the node, followed by “immunotherapy” and “melanoma”. As shown in **Figure 7A**, all identified keywords can be divided into 3 categories, “ocular melanoma immunotherapy research” “ immune checkpoint research” and “immune checkpoint inhibitors research”. These clusters are the most prominent topics in ocular melanoma immunotherapy at present. For the “ocular melanoma immunotherapy studies” cluster, the main keywords are uveal melanoma, immunotherapy, mucosal melanoma, conjunctival melanoma, prognosis, metastatic uveal melanoma, and tumor microenvironment. The main keywords clustered in the “immune checkpoint studies” were melanoma, targeted therapy, braf, bap1, metastasis, and nras. While in the “immune checkpoint inhibitors research”, the main keywords were ipilimumab, pd-1, ocular melanoma, pembrolizumab, and nivolumab. The density visualization of keywords is shown in **Figure 7B**, the top three most frequent keywords are “uveal melanoma”, followed by “immunotherapy” and “melanoma”. The overlay visualization map is shown in **Figure 7C**, which summarizes the occurrence of keywords from the perspective of time zones. Burst keywords are terms that are frequently cited over a while. The burst keywords were detected using the CiteSpace algorithm. The top 24 keywords with the highest burst intensity are shown in **Figure 7D**. The keyword with the highest citation frequency was “uveal melanoma” (2017-2021), followed by “immunotherapy “(2017-2021) and “melanoma” (2015-2020). The keywords with the longest outbreak were “metastases”, which lasted 16 years from 2002 to 2018. In particular, “bap1”, “tebentafusp”, “bioinformatics”, “ conjunctival melanoma”, and “immune checkpoint inhibitors” are five keywords that are still in the process of explosion. Burst keywords are words that are frequently cited over a period of time. The top 10 keywords with the strongest citation bursts are shown in **Figure 7E**. The blue line represents the period from 1991 to 2022, while the period of each burst keyword is plotted by the red line. Keywords with burst citations after 2014 are “ ipilimumab” (2014-2019, intensity of 6.41), “pembrolizumab” (2017 -2019, intensity of 5.7), “pd 1” (2019-2020, intensity of 4.51), “tumor-infiltrating lymphocyte” (2019-2020, intensity of 4.41), “immune checkpoint inhibitor” (2020-2022, intensity 4.73). In particular, the keyword “immune checkpoint inhibitor” is still in the process of explosion.

## 5 Discussion

### 5.1 The trend overview

This study was the first to use a bibliometric approach to measure research trends in immunotherapy for ocular melanoma from 1991-2022. Bibliometric analysis is now a powerful tool for summarizing the current state of knowledge and predicting future trends, and visual maps are generated using VOSviewer or CiteSpace (33, 35) to show specific knowledge domains and structural relationships. This study shows a gradual increase in the number of publications per year from January 1, 1991, to October 19, 2022 (**Figure 2**), indicating an increase in interest in this field as well. In terms of country contribution, as shown in **Table 1** and **Figure 3**, the United States is the most prolific and leading country in this field, with the largest number of publications, a wider total citation frequency, and the largest H-index. The U.S. has some of the best researchers and institutions in the world and is a leader in the field of immunotherapy for ocular melanoma. With the economic development of China, the rising demand for healthcare in China as a populous country, and the gradual increase in financial support, the interest in molecular biotherapeutics has also increased. However, China has the second highest number of papers in the world, the total citation frequency, average citation frequency, and H-index are low. Despite the rapid economic development in China, the development of the biomedical field is relatively lagging behind with a weak foundation. In addition, the high cost of medical care in China as a developing country is a challenge for health insurance, which also limits the clinical promotion as well as the development of immunotherapy to some extent (36, 37). Among the research institutions, the UNIVERSITY OF TEXAS SYSTEM (36 articles), MEMORIAL SLOAN KETTERING CANCER CENTER (32 articles), and LEIDEN UNIVERSITY (31 articles) have made positive contributions to the research frontier. It is noteworthy that the top 5 research institutions have significantly increased the number of publications in the ocular melanoma research, which is largely consistent with the number of papers published globally by the top 5 countries, indicating the dominant role of top-tier institutions in improving a country’s academic research ranking. Thus, this evidence also suggests that further in-depth collaborative research may play a crucial role in research related to the immunotherapy of ocular melanoma, guiding future investigators to publish high-quality papers. The research output of these countries may be associated with leading pioneering researchers in the field and substantial financial support. Indeed, the top authors and co-cited authors are mainly from Europe and the United States (**Table 3**, **Table 4**). The top-ranked authors with the most publications listed in **Table 3** are relatively early entrants who may be prioritizing new advances in immunotherapy related to ocular melanoma. The most published journal in this field was CANCERS (IF=6.575,2021), followed by MELANOMA RESEARCH (IF=3.199,2021) and FRONTIERS IN ONCOLOGY (IF=5.738,2021). Due to the rare nature of ocular melanoma, the selection of journals is narrow. Researchers can follow these journals to find out about the latest developments in immunotherapy for ocular melanoma. Additionally, future researchers may consider publishing their high-quality findings in the top 10 journals.

### 5.2 Research hotspots and frontiers

Research frontiers and hotspots in a particular field of study might be reflected in keywords. By keyword co-occurrence analysis, the keywords in WoSCC were divided into 3 clusters, “ ocular melanoma immunotherapy research “ “ immune checkpoint research” and “immune checkpoint inhibitors research”. These 3 clusters represent the main research directions of ocular melanoma. Based on the overlay visualization in **Figure 7C**, it can be concluded that the research hotspots in this field have gradually shifted from “immune checkpoint research” to “immune checkpoint inhibitors research. For “ ocular melanoma immunotherapy research “, the research hotspots have gradually shifted from “uveal melanoma research” to “metastatic uveal melanoma research” and “conjunctival melanoma research”, and “tumor microenvironment research”. This change in the field is in line with the development of translational medicine, indicating that basic research on immunotherapy of ocular melanoma has become more mature, and multiple immune targets have been identified. With the translation of basic research to the clinic and the increased investment in new drug research, researchers are gradually focusing their research on clinical studies. There are still several keywords in the outbreak, indicating that immunotherapy of ocular melanoma remains a hot spot for research. There is a need to strengthen research in this area to provide more treatment options for patients with different types of ocular melanoma and to promote individualized and precise treatment. In addition, combining several keywords with the highest outbreak intensity, we speculate that “bap1”, “tebentafusp” and “bioinformatics” may become a hot research topic in the coming years.

#### 5.2.1 Advances in the molecular mechanisms of ocular melanoma

The analysis of co-citation clusters helps us to understand the dynamic evolution of research related to immunotherapy of ocular melanoma over the last 30 years. Cluster 1 (nras) and cluster 6 (diagnosis) were initiated early, suggesting that they are the basis of research on immunotherapy of ocular melanoma. Ocular and cutaneous melanocytes are functionally similar and have the same embryonic origin, but undergo different transformations during tumorigenesis (38, 39). Cutaneous melanoma is mainly triggered by mutations in proto-oncogene neurofibromin 1 (NF1), neuroblastoma RAS viral oncogene homolog (NRAS), and BRAF gene of serine/threonine-protein kinase BRAF (40–42). In conjunctival melanoma, its activation is most often dependent on BRAF, NRAS, or KIT mutations. The frequency of BRAF, NRAS and KIT mutations in conjunctival melanoma is more similar to that in cutaneous melanoma than in uveal melanoma (43, 44), which is consistent with **Figure S1**, whereas oncogenic driver mutations in uveal melanoma are present in paralogous guanine nucleotide-binding protein Gq subunits alpha and subunit alpha-11 (respectively GNAQ and GNA11) in the genes of the downstream Hippo/YAP (Yes-associated protein) and RAS/mitogen-activated protein kinase (MAPK) signaling pathways have been shown to contribute to the development and progression of uveal melanoma (9, 45–48). BAP1 is a tumor suppressor gene that encodes a nuclear deubiquitinase involved in cell growth and cancer pathogenesis and maps to chromosome 3 (49). BAP1-inactivating mutations are found in approximately 47% of primary uveal melanoma and 84% of metastatic uveal melanoma cases, consistent with an association between BAP1 mutations and poor prognosis (50). Chromosome 3 loss has long been the strongest indicator of metastatic disease in UM patients (51). Combined with our data, biomarkers represented by bap1 will be the future hot spot for immunotherapy of ocular melanoma. Dono et al. analyzed 50 cases of primary uveal melanoma obtained after excision with the gene mutations GNAQ, GNA11, and BAP1, They found that 42.2% of uveal melanomas contained mutated GNAQ, 32.6% GNA11, 31.5% BAP1, 9.7% SF3B1, 18.9% EIF1AX and 1% TERT, where GNAQ and GNA11 were usually mutually exclusive, but both could coexist with BAP1 or SF3B1 mutations. Similarly, BAP1 and SF3B1, EIF1AX, and SF3B1 mutations are mutually exclusive, and TERT mutations appear to specifically coexist with GNA11 or EIF1AX mutation (52–54). These molecular factors are involved in the development of ocular melanoma and serve as potential therapeutic targets for immunotherapy, which should be further explored in the future. The ongoing explosion of cluster 7 (bioinformatics) also validates this speculation.

#### 5.2.2 Advances in immunotherapy for ocular melanoma

Despite definitive initial treatment and aggressive surveillance for ocular melanoma, up to 50% of patients will develop metastatic disease. The standard of care for patients with primary uveal melanoma after definitive therapy is to expect observation or participation in clinical trials. Cluster 8 (dacarbazine), on the other hand, represents an initial exploration into the treatment of metastatic ocular melanoma. Dacarbazine is an anti-cancer drug known as an alkylating agent that kills cancer cells by adding an alkyl group to their DNA (55, 56). Dacarbazine is a single FDA-approved anticancer drug that is now used as the chemotherapy drug of choice for the treatment of melanoma (57, 58). As research progresses, there is also increasing evidence that certain chemotherapeutic agents have broader activity and that they should also be considered immunomodulators. Hervieu demonstrated that dacarbazine exerts an immunostimulatory effect by inducing local activation of natural killer cells and T cells, suggesting that the tumor is involved in the initiation of the immune response during treatment with dacarbazine (59). Considering the role of dacarbazine as an immunomodulator, dacarbazine may be able to be used in combination with immunotherapeutic agents in the treatment of ocular melanoma (60–62).

The eye is an immune-privileged region of the body and is associated with multiple immunosuppressive mechanisms (63, 64). It is thought that ocular melanoma may be highly immunogenic when cells are systemically dispersed and hence may be vulnerable to immune checkpoint inhibition since it has mechanisms to elude the immune system (65, 66). Uveal melanoma can downregulate major compatibility complex I (MHC) molecules and block the recruitment and activation of cytotoxic t lymphocytes (CTL) (63), and once the ocular melanoma metastasizes or becomes infiltrated with inflammation, the expression of MHC I is elevated (67, 68). In addition, tumors impede immune responses by increasing the secretion of suppressive cytokines such as tumor growth factor (TGF)-b, the activation of suppressive cell types such as regulatory T cells, and the activation of suppressor receptors such as T lymphocyte-associated protein 4 (CTLA-4) and programmed cell death protein 1 (PD- 1) (69–71). These findings all suggest that the above molecules may be potential targets for therapy.

In recent years, therapeutic options to overcome tumor immunosuppressive mechanisms be effective in the treatment of cutaneous melanoma (72). These approaches have also been applied at ocular melanoma (73). Cluster 3 (ipilimumab) is a humanized monoclonal antibody against CTLA-4 that blocks the immunosuppressive interaction between CTLA-4 and B7 (74, 75). Ipilimumab was first approved by the FDA in 2011 for the treatment of metastatic or unresectable cutaneous melanoma that has received at least one prior therapy and is now approved for first-line and adjuvant treatment of advanced melanoma (76–78). A portion of clinical studies for immunotherapy of ocular melanoma has been conducted. The efficacy and tolerability of ipilimumab were retrospectively evaluated in 104 patients with melanoma in Australia with a median follow-up of 7 months and a median OS of 9.6 months (95% CI, 6.6 ~ 12.4). The study found that the median OS in patients with non-cutaneous (mucosal and uveal) melanoma (n = 11) was almost half that of patients with cutaneous melanoma (n = 79): 5.8 months (95% CI, 2.8-12.4) versus 11.7 months (95% CI, 7.1-13.8) with similar PFS periods (79). In a study of ipilimumab in 39 patients with UM, a sustained complete response was found in 1 patient after 62 weeks of treatment and a delayed partial response in 1 patient (100 weeks after stable disease); median overall survival was 9.6 months (95% CI 6.3 ~ 13.4 months) (80). Another potential therapeutic approach for ocular melanoma is to overcome the immunosuppressive mechanism by disrupting PD-1/PD-L1 ligand-receptor interactions with specific antibodies (81, 82). The anti-PD-1 antibodies nivolumab and pembrolizumab and the anti-PD-L1 antibody atezolizumab have been approved for the treatment of melanoma (83–86). Only a few individuals have received treatment for conjunctival melanoma with PD-1 inhibitors alone, CTLA-4 inhibitors alone, or a combination of the two. There have been numerous reports of high complete and partial remission rates (87, 88).

Cluster 0 (tebentafusp) has the largest node and is also a hot spot for future research in ocular melanoma immunotherapy. Tebentafusp is novel immunotherapy based on the immune-mobilizing monoclonal T cell receptor against cancer (ImmTAC) platform. Tebentafusp targets the HLA-A*02:01 presented with a fragment of the melanocyte spectrum-specific antigen gp100 280-288 (also known as melanocyte protein Pmel17, melanoma-associated ME20 antigen, ME20-m) (89, 90). Gp100 is strongly expressed in melanoma cells, weakly expressed in normal melanocytes, and minimally expressed in other tissues (90, 91). Based on small clinical studies, tebentafusp showed promising clinical activity in patients with metastatic UM, and its survival appears to be superior to that reported with other treatments (92, 93). In view of the current state of research on ocular melanoma, we suggest that future studies should focus on more systematic prospective studies to gain a comprehensive understanding of the treatment strategies and prognosis of ocular melanoma with different genetic bases.

## 6 Limitations

We believe this is the first study to use bibliometric techniques to summarize the development and current status of immunotherapy for ocular melanoma. There are still certain restrictions that need to be researched, though: (1) Selection bias in databases: The whole body of literature included in this investigation was acquired from WoSCC and Pubmed. It’s possible that pertinent studies from other databases were left out. (2) Because non-English or non-research/review papers were not included in this study, some omissions may have occurred. We only extracted research and review articles in English. (3) Because research are updated regularly, it’s possible that we missed some recently published, significant studies.

## 7 Conclusion

In summary, this study is the first scientific and comprehensive analysis of global research trends in immunotherapy for ocular melanoma over the past 30 years using a bibliometric approach. This study systematically summarizes global publication trends in the field and helps scholars identify key authors, institutions, and journals in the field. Keyword and co-citation cluster analyses also guide researchers to select new research directions. In order to direct future research paths in immunotherapy, there is an urgent need to investigate novel target molecules and carry out high-quality randomized controlled studies on current immune drugs.

## Data availability statement

The original contributions presented in the study are included in the article/**Supplementary Material**. Further inquiries can be directed to the corresponding authors.

## Author contributions

YT and YL contributed to the study design. YT and YL collected and cleaned the data. YT and YL wrote the original manuscript. YT and YL participated in the statistical analysis. BQ revised the article. All authors read and approved the final version of the manuscript. All authors contributed to the article and approved the submitted version.
