# Supplementary material for: Immunotherapy for ocular melanoma: a bibliometric and visualization analysis from 1991 to 2022
Source: Front Oncol. 2023 May 31;13:1161759. doi: 10.3389/fonc.2023.1161759 (PMC10265996; doi:10.3389/fonc.2023.1161759)
Supplement: Supplementary file 1 [file DataSheet_1.docx]

Supplementary Material

# Supplementary Data

## This study identifies search strategies for articles from the Clarivate Analytics Web of Science Core Collection database.

**#1 :** TS=(“uveal melanoma*” OR “ocular melanoma*” OR “choroidal melanoma*” OR “choroidal metastases” OR “iris melanoma*” OR “conjunctival melanoma*” OR “ciliary body melanoma*” OR “Melanoma of the Uvea” OR “Uveal Neoplasms” OR "orbital melanoma*").

**#2 :** TS=(“Immunotherap*” OR “Immune Checkpoint Inhibit*” OR “Immune Checkpoint Block*” OR “targeted therapy” OR “tebentafusp” OR “IMCgp100” OR “ipilimumab” OR “nivolumab” OR “pembrolizumab” OR “dendritic cell vaccine” OR “glembatumumab vedotin” OR “atezolizumab” OR “tremelimumab” OR “MELITAC 12.1” OR “epacadostat” OR “PD-L1 Inhibit*” OR“PD-L1 Block*” OR “PD L1 Inhibit*” OR“PD L1 Block*” OR “Programmed Death-Ligand 1 Inhibit*” OR “Programmed Death Ligand 1 Inhibit*” OR “PD-1 Inhibit*” OR “PD-1 Block*” OR “PD 1 Inhibit*” OR “PD 1 Block*” OR “Programmed Cell Death Protein 1 Inhibit*” OR “Programmed Cell Death Protein 1 Block*” OR “PD-1-PD-L1 Block*” OR “PD-1-PD-L1 Inhibit*” OR “PD 1 PD L1 Block*” OR “PD 1 PD L1 Inhibit*” OR “CTLA-4 Inhibit*” OR “CTLA-4 Block*” OR “CTLA 4 Inhibit*” OR “CTLA 4 Block*” OR “Cytotoxic T-Lymphocyte-Associated Protein 4 Inhibit*” OR “Cytotoxic T-Lymphocyte-Associated Protein 4 Block*” OR “Cytotoxic T Lymphocyte Associated Protein 4 Inhibit*” OR “Cytotoxic T Lymphocyte Associated Protein 4 Block*” OR “Checkpoint Inhibit*” OR “Checkpoint Block*” OR “PD-L1” OR “PD-1” OR “CTLA-4” OR “PD L1” OR “PD 1” OR “CTLA 4”).

**#3 :** #1 AND #2.

Indexes=SCI-EXPANDED.

Timespan= From January 1991 to October 2022.

Note: SCI-EXPANDED, Science Citation Index Expanded.

## This study identifies the search strategy for articles from the Pubmed database.

**#1 :** “uveal melanoma*[Title/Abstract]” OR “ocular melanoma*[Title/Abstract]” OR “choroidal melanoma*[Title/Abstract]” OR “choroidal metastases[Title/Abstract]” OR “iris melanoma*[Title/Abstract]” OR “conjunctival melanoma*[Title/Abstract]” OR “ciliary body melanoma*[Title/Abstract]” OR “Melanoma of the Uvea[Title/Abstract]” OR “Uveal Neoplasms[Title/Abstract]” OR "orbital melanoma*[Title/Abstract]".

**#2 :** “Immunotherap*[Title/Abstract]” OR “Immune Checkpoint Inhibit*[Title/Abstract]” OR “Immune Checkpoint Block*[Title/Abstract]” OR “targeted therapy[Title/Abstract]” OR “tebentafusp[Title/Abstract]” OR “IMCgp100[Title/Abstract]” OR “ipilimumab[Title/Abstract]” OR “nivolumab[Title/Abstract]” OR “pembrolizumab[Title/Abstract]” OR “dendritic cell vaccine[Title/Abstract]” OR “glembatumumab vedotin[Title/Abstract]” OR “atezolizumab[Title/Abstract]” OR “tremelimumab[Title/Abstract]” OR “MELITAC 12.1[Title/Abstract]” OR “epacadostat[Title/Abstract]” OR “PD-L1 Inhibit*[Title/Abstract]” OR“PD-L1 Block*[Title/Abstract]” OR “PD L1 Inhibit*[Title/Abstract]” OR“PD L1 Block*[Title/Abstract]” OR “Programmed Death-Ligand 1 Inhibit*[Title/Abstract]” OR “Programmed Death Ligand 1 Inhibit*[Title/Abstract]” OR “PD-1 Inhibit*[Title/Abstract]” OR “PD-1 Block*[Title/Abstract]” OR “PD 1 Inhibit*[Title/Abstract]” OR “PD 1 Block*[Title/Abstract]” OR “Programmed Cell Death Protein 1 Inhibit*[Title/Abstract]” OR “Programmed Cell Death Protein 1 Block*[Title/Abstract]” OR “PD-1-PD-L1 Block*[Title/Abstract]” OR “PD-1-PD-L1 Inhibit*[Title/Abstract]” OR “PD 1 PD L1 Block*[Title/Abstract]” OR “PD 1 PD L1 Inhibit*[Title/Abstract]” OR “CTLA-4 Inhibit*[Title/Abstract]” OR “CTLA-4 Block*[Title/Abstract]” OR “CTLA 4 Inhibit*[Title/Abstract]” OR “CTLA 4 Block*[Title/Abstract]” OR “Cytotoxic T-Lymphocyte-Associated Protein 4 Inhibit*[Title/Abstract]” OR “Cytotoxic T-Lymphocyte-Associated Protein 4 Block*[Title/Abstract]” OR “Cytotoxic T Lymphocyte Associated Protein 4 Inhibit*[Title/Abstract]” OR “Cytotoxic T Lymphocyte Associated Protein 4 Block*[Title/Abstract]” OR “Checkpoint Inhibit*[Title/Abstract]” OR “Checkpoint Block*[Title/Abstract]” OR “PD-L1[Title/Abstract]” OR “PD-1[Title/Abstract]” OR “CTLA-4[Title/Abstract]” OR “PD L1[Title/Abstract]” OR “PD 1[Title/Abstract]” OR “CTLA 4[Title/Abstract]”.

**#3 :** #1 AND #2.

# Supplementary Figures and Tables

## **Supplementary Figures**

**Figure S1. The timeline view of clusters of co-cited references of conjunctival melanoma using by CiteSpace.**

**
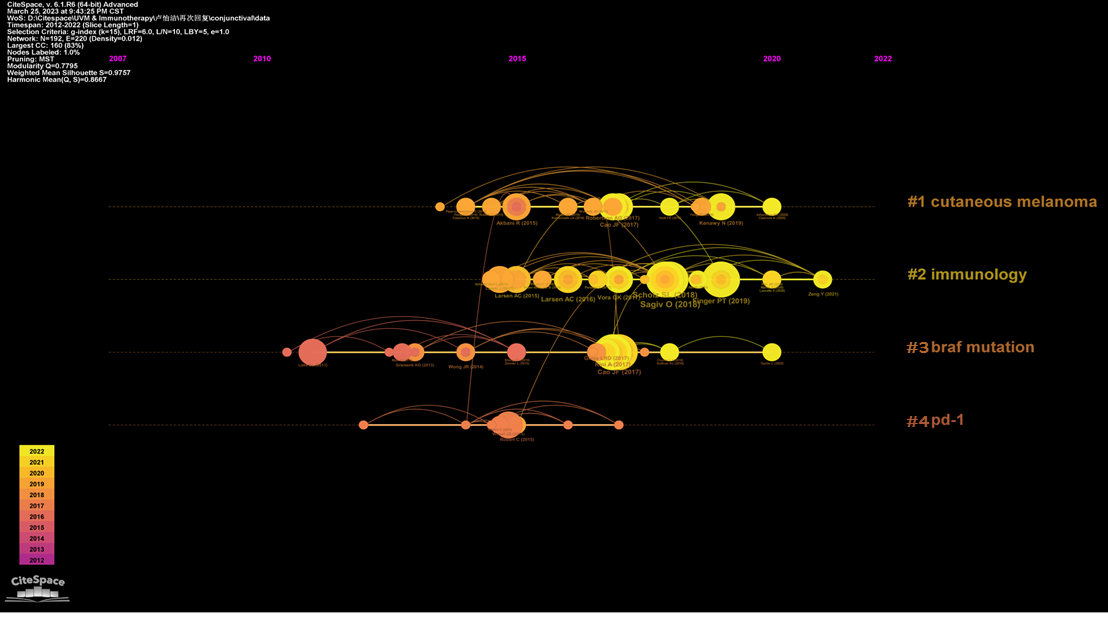
**

## Supplementary Tables

**Table S1.** **Keyword co-occurrence network cluster table.**

| **Cluster ID** | **Size** | **Silhouette** | **Mean**  **(Year)** | **Top Terms (LSI)** | **Top Terms (LLR)** | **Top Terms (MI)** |
| --- | --- | --- | --- | --- | --- | --- |
| 0 | 52 | 0.955 | 2018 | uveal melanoma; immune checkpoint blockade | tebentafusp; braf; | combined immune checkpoint blockade; percutaneous hepatic perfusion |
| 1 | 39 | 0.971 | 2011 | targeted therapy; nras | nras; inhibitor; | autoimmunity; melanocytic nevus |
| 3 | 34 | 0.929 | 2017 | uveal melanoma; DNA methylation | ipilimumab; therapy | macrophage; tumor immune microenvironment |
| 4 | 34 | 0.956 | 2015 | uveal melanoma; immune checkpoint blockade | mucosal melanoma;  t-vec | sotrastaurin; ezh2 |
| 5 | 33 | 0.942 | 2012 | targeted therapy; glembatumumab vedotin | uveal melanoma; nras | medical resource utilization; braf inhibitors |
| 6 | 30 | 0.975 | 2009 | uveal melanoma; new therapeutic agents | diagnosis; conjunctival | conjunctival; enucleation |
| 7 | 29 | 0.992 | 2018 | uveal melanoma; cancer vaccine | bioinformatics; melanoma | infiltrating immune cells; adaptive immune resistance |
| 8 | 28 | 0.978 | 2013 | uveal melanoma; immune checkpoint blockade | dacarbazine; ipilimumab | cytotoxic t-lymphocyte-associated antigen-4; acral cutaneous melanoma |
| 9 | 27 | 0.995 | 2015 | uveal melanoma; immune checkpoint inhibitors | nras; melanoma | immtac platform; t cell receptor |
